# Interview with Lord Jonathan Sumption

**DOI:** 10.1192/bjb.2024.47

**Published:** 2024-08

**Authors:** Abdi Sanati

**Affiliations:** Meets former Justice of the Supreme Court of the UK and eminent historian Lord Jonathan Sumption

**Figure:**
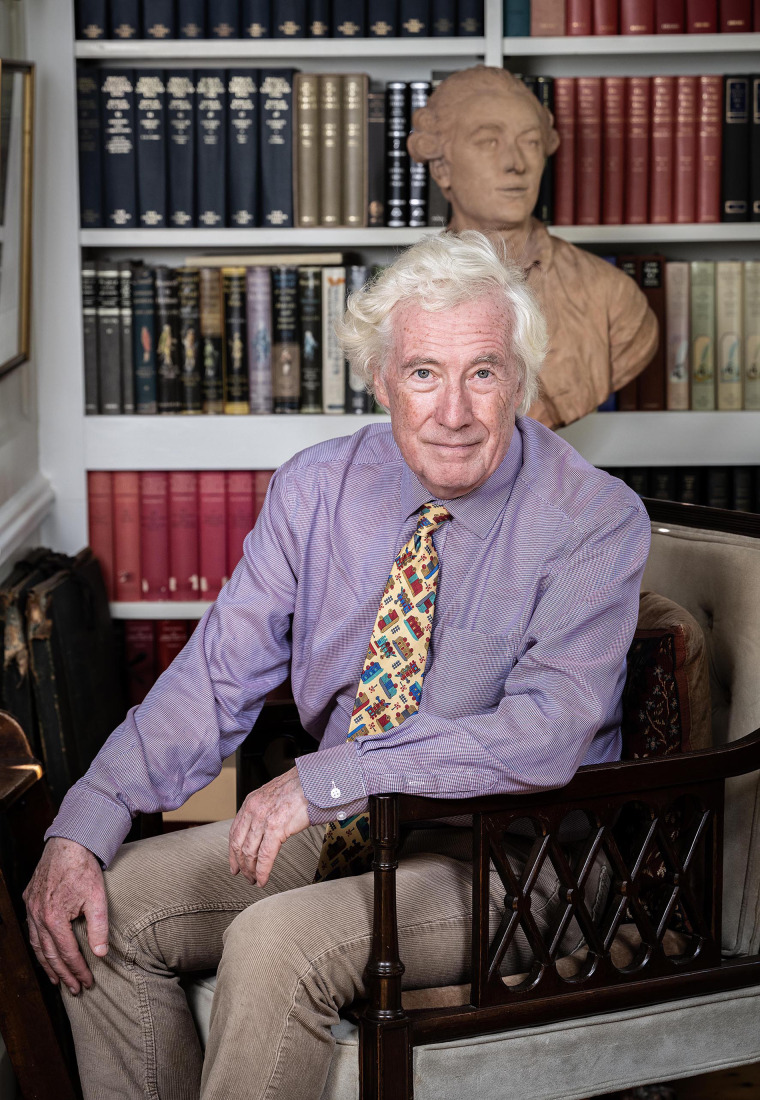

Lord Jonathan Sumption is a former Justice of the Supreme Court of the UK. As well as his distinguished career in the Judiciary, Lord Sumption is an eminent historian with many publications, including a five-volume history of the Hundred Years’ War. I have been following Lord Sumption's articles in papers and admired his sharpness of mind and clarity of writing. I was fortunate to have the opportunity to interview Lord Sumption for the *BJPsych Bulletin*.

**Many thanks for the opportunity Lord Sumption. I recently read your article on assisted dying. I really enjoyed the clarity of your arguments, especially when you explained the opposing values of life and autonomy and how it's difficult to reconcile them at a conceptual level. I wonder if you could give the readers a summary of your arguments please.**

I became interested in the whole issue of assisted dying as a result of sitting on the Supreme Court in the case of Nicklinson, which is the leading case on the subject. Indeed, I wrote one of the principal judgments. The decision was inconclusive. My view was that the issue was not a suitable one to be decided by courts at all, and that was the majority view, albeit for different reasons. The result was that the Court declined to make a declaration that there was a human right to medical assistance in suicide or that the current law was incompatible with the Human Rights Convention. I remained neutral, varying on either side of the neutral line for quite some time after that. I think the arguments are very nicely balanced. But I have recently come to the view, very much on balance, that there is a case for allowing medically assisted suicide in the case of terminally ill patients. The case is based on the same argument that commended itself 2000 years ago to the Roman philosopher Seneca, who said that at the end of life, if you deny people the right to commit suicide, you are not prolonging life. You are just prolonging the process of dying. I think that's a point that has considerable force, particularly in the case of people who are faced with a painful, unpleasant and slow death. The contrary argument is that if we normalise suicide as an exit route, it will be chosen for much less weighty reasons. That is a particular risk in our society, which has very negative views about old age. I don't think that there is a serious risk that unscrupulous heirs will deliberately encourage old people to take their own lives. But I do think that there is a serious risk that at the end of their lives, people will undervalue themselves. They will feel that they are a burden to their relatives. They will be encouraged by the mood around them to feel that they have a duty to do away with themselves in order to relieve the relatives of that burden. I think that is a very serious risk. I've heard it said that there's nothing wrong with that. I think that there is. I think that when people start to consider suicide on the basis of a view which they feel that society holds about them, we are heading towards a pretty unpleasant place. Nonetheless, I favour a compromise solution, which I think is acceptable, that those who are terminally ill should be allowed to have medical assistance in committing suicide just as they currently enjoy the right to medical assistance in palliating pain and in explaining to them what the options are.

**In some countries, like Belgium, and it might happen in Canada, they are bringing assisted dying for the mentally ill. What is your opinion?**

The problem about mental illness is that it undermines one's capacity to make any decision at all. The first requirement for recognising any right to medical assistance in suicide is that the patient should be of sound mind and fully capable of making an observant and rational decision about his or her life. In the case of the mentally ill, that's a test which is much more difficult to satisfy.

**You briefly mentioned rights. And the concept of rights has been very dominant in our society. You also have a chapter about it in your book (*Trials of the State*).^1^ The concept of rights is very big in psychiatry. What are your views on morality and law that are based on rights?**

Well, that's a very large subject. Obviously, the law, especially the civil law, is built upon a scheme of rights. We have rights in contract, tort and so on. But human rights are a rather different sort of creature. They are essentially rights against the public and against the state, to have the law take a particular form. In a democracy it is absolutely essential that rights should ultimately be decided by representative bodies, by Parliament, and that is essentially a political process. My objection to the current use of human rights is that it essentially diverts what are really political issues from the sphere of politics to the sphere of law, and therefore transfers responsibility from legislatures to the courts. When you do that, you marginalise the views of the public, who have no influence, quite rightly, over the work of the courts but are entitled to a significant say in the work of the legislature. I think that human rights are a fundamentally autocratic and anti-democratic process. Indeed, they will be rationalised by very many advocates of human rights as being necessary, precisely because these people do not trust democratic decision-making. They fear that democratically mandated decisions may be oppressive to minorities, or otherwise irrational or illiberal. And sometimes they may, but I don't think it's a strong argument against democracy, to say that the people may choose policies that the speaker doesn't like.

**Reading about human rights, I couldn't help but think of what happens in the USA. They have this constitutional rights concept and everything they want, they phrase it in terms of constitutional rights. In Britain, we don't have that constitution, which I think is good, because these things are very abstract. Could they ever be useful in a way that they were intended to be?**

We can have whatever rights we like, as a matter of domestic legislation. There isn't a single thing in the European Convention on Human Rights which we cannot have, through our own ordinary democratic decision-making processes, if we want it. The purpose of the Human Rights Convention, which is essentially an international treaty enforced by an external court, is to make us accept certain rights whether we want them or not.

It is expressly justified as a limitation on democracy. I think that there are perfectly legitimate reasons for limiting democracy but not in that particular way. The main method of limiting democracy is indirect representation, whereby we do not have referenda on every issue. We elect people whose wisdom we trust, and if we don't like what they do, we can get rid of them.

**I read recently, you argued for leaving the European Court of Human Rights (EHCR). Do you still advocate Britain leaving the ECHR?**

Yes, I have come to that view relatively recently. For some years, I thought although the Human Rights Court in Strasbourg was a very unsatisfactory body, it was more satisfactory to try to reform it from the inside. I've come to the conclusion that it's not capable of being reformed from the inside. It is an ideologically committed court. Ideologically committed bodies are not easy to reform from the inside or indeed from any direction at all. I would not favour dumping the whole concept of human rights. I would favour re-enacting the human rights convention as an English statute and withdrawing from the Human Rights Convention. That would mean that we would effectively get rid of the Strasbourg Court but retain the rights in the text of the Convention, most of which existed in law in Britain for many, many years before the Human Rights Act. They are in themselves unobjectionable. What is objectionable is the vast expansion of their scope in order to cover many things that were not intended in the Convention. This confers on the Strasbourg Court what is in reality a legislative power. In a democracy, I do not think that legislative powers should be conferred on external bodies over whom the electorate has no influence, direct or indirect.

**In your book, you explain the judicial overreach. When it comes to the Strasbourg Court, recently we had this judgment on climate change against Switzerland.**

It is a very interesting indicator of the direction in which we are moving – because the judgment is a direct assault, in my view, on democracy and indeed on sound governments, and I'll explain why. It concerns the climate change legislation of Switzerland. In 2020, the Swiss enacted a very stringent climate change law, which set out targets that were regarded as too exacting by the Swiss electorate. Under the Swiss constitution, provided that you can get together a sufficient number of signatures, you can require a referendum on any Act of the Swiss Parliament. A referendum was required on this Act, and the Act failed. A majority were opposed to it. So the Swiss Parliament enacted a new, more moderate Act, which is actually strikingly similar in terms to the relevant English legislation passed in 2008. It was accepted in a referendum the following year. The Strasbourg Court has basically held that the electorate was not entitled as a matter of human rights law to reject the earlier Act. They object to the new Act on the grounds that the intermediate stages of emissions reduction for which it provides were not ambitious enough; and that too much discretion, particularly as to timing, was left to the Swiss government and parliament; and that it doesn't specify exactly what measures they are required to take to achieve the targets. It also objects to the use of qualifying phrases like ‘as far as possible’, which they say is not sufficiently absolute. Now, the question which immediately comes to mind when you read a judgment like that is: what place is there in the scheme of things for democracy? Switzerland is probably the most democratic country in the world, operating by a combination of referenda and parliamentary legislation. In the relevant paragraph of the Strasbourg Court's judgment, what it says is, well, democracy isn't just a matter of majorities. If democracy is not a matter of majorities, I am at a loss to know what it is. What they say is that majorities must always yield to the rule of law. There was nothing in the Swiss government's and the Swiss Parliament's behaviour that was contrary to the rule of law. What the Strasbourg Court meant by the rule of law was the decisions of the Strasbourg Court. So essentially what the Strasbourg Court is saying in that critical paragraph is that democracy must always yield to the opinions of the Strasbourg Court. Now, the opinion of the Strasbourg Court about what was required in this instance was based on Article 8. Article 8 protects the right to privacy and family life and the security of correspondence. It has nothing whatever to do with climate change. The Strasbourg Court has expanded the scope of private and family life to cover absolutely every aspect of human well-being. In other words, anything, or almost anything. So by treating Article 8 as a licence to intervene in any subject whatever, the Strasbourg Court has arrogated to itself the right to determine the laws of the 46 countries of the Council of Europe, without reference to the wishes of their democratic electorates. To my mind, that is completely objectionable. It is not only undemocratic. It is also contrary to sound government, because the problem about courts is that they are not capable of dealing with polycentric issues. The Strasbourg Court was only dealing with climate change. Politicians do not have the luxury of thinking about one thing at a time. Politicians have to look at the issue in the round. They have to look at an issue like climate change in the light of all the other policy considerations and in the light of the impact of the measures required to deal with it on their populations. The populations of Europe have built their lives on the basis of past attitudes to the availability of energy. They have houses to heat. They have work to get to, which may be somewhere only accessible by road. They have, in many cases, very tight budgets. It is not possible to ride roughshod over the population at large. Politicians have to find a compromise which works but at the same time addresses legitimate interests of the population other than climate change. To have a system of adjudication which looks at only one issue at a time and not at its practical implications is, to my mind, a defiance of basic principles of good government.

**Going back to your book, you mentioned that when politicians don't do their job and there's a vacuum, sometimes the law comes to fill that vacuum. In recent years I've noticed that apart from the judiciary, some designated experts also come to fill this vacuum. We actually saw it in COVID that that the politicians delegated their role to the experts in name of ‘following the science’.**

I don't think there was a vacuum in that case. What happened was politicians wishing to cover their backs by claiming to be following the science. This was basically an attempt by those in power to pass the responsibility to others. So I don't think there was actually a vacuum there at all.

**I did admire your stance on lockdown. Mentioning anything against lockdown was like committing a heresy, and it did result in some negative consequences. The lockdown had a direct negative impact on mental health which is not talked about even now.**

They're talking about it more nowadays. We are coming to realise the catastrophic implications that lockdowns had for mental health, particularly among the young. I think that this is probably closely connected to the disappearance from the workforce of a very large number of people, most of them young. This has been a great misfortune. My feeling is that we are coming to realise this; whether it will be realised by Lady Justice Hallett, who's conducting the inquiry currently in progress, is another question. Perhaps not.

**In your book, you mentioned that when a clinician takes a matter to court they want the court to make a decision which is basically clinical. I have seen it a lot and I think we have become very risk averse.**

I don't entirely blame the clinicians, because we live in a very litigious society, and the courts have a power of absolution. So if you go and ask the court's permission to do something and they give it, you are protected against litigation. It's partly risk aversion. But it's also a feeling among the public that for every misfortune there is a governmental solution. That is fundamentally false. There are many things which are beyond the powers of social institutions and beyond the powers of governments. Nowadays, it is extremely difficult for a death to occur, other than in extreme old age, without people feeling that something was wrong; that someone slipped up, that some one must be held to account. We are not willing to accept the implications of ill health and mortality. We wish to believe that there is a system that will do away with these things. This is an illusion. It's a very dangerous illusion, because it inevitably leads to disappointment and frustration. This is what leads people to be far too ready to sue. That's why clinicians go to the court for authority to do anything controversial whenever they can.

## References

[1] Sumption J. Trials of the State: Law and the Decline of Politics. Profile Books, 2019.

